# Differences in orexin-A level in the functional brain network of HUD patients undergoing harm reduction therapy

**DOI:** 10.1097/MD.0000000000030093

**Published:** 2022-08-19

**Authors:** Wan-Ru You, Liang-Chun Lin, Wei-Che Lin, Meng-Chang Tsai

**Affiliations:** a Department of Psychiatry, Kaohsiung Chang Gung Memorial Hospital, and Chang Gung University College of Medicine, Kaohsiung, Taiwan; b Department of Radiation Oncology, China Medical University Hospital, Taichung, Taiwan; c Department of Diagnostic Radiology, Kaohsiung Chang Gung Memorial Hospital, and Chang Gung University College of Medicine, Kaohsiung, Taiwan.

**Keywords:** functional brain network, harm reduction therapy, heroin, HUD, orexin-A

## Abstract

Orexins regulate the reward-seeking pathway and also play a role in drug addiction. The aim of this study was an investigation of the changes in serum level of orexin-A as well as changes in the functional brain network in heroin use disorder (HUD) patients undergoing harm reduction therapy (HRT).

Twenty-five HUD patients undergoing HRT that included methadone and buprenorphine, and 31 healthy control (HC) subjects, were enrolled for this study. Serum orexin-A levels and brain-derived neurotrophic factor were measured with assay kits. The functional brain network in HUD patients and HC was investigated and assessed using seed-based analysis and functional brain MRI scans.

*t* Tested orexin-A levels were found to be significantly higher in HUD patients undergoing HRT than in HCs (*P* < .05). Analysis showed the functional activity of the right ventral anterior insula (RVAI) in HUD patients to be significantly lower than in HCs (*P* < .05, Family-Wise Error) corrected). In addition, the internetwork functional connectivity was significantly lower in the left nucleus accumbens and left dorsal anterior insula in the HUD subjects than in HCs (*P* < .05, Family-Wise Error corrected).

In this study, no significant correlation between orexin-A levels and functional brain networks was found. However, the results suggest that HRT might increase orexin-A levels and decrease functional activity in RVAI in HUD patients.

## 1. Introduction

Orexins play a role in the reward-seeking pathway. This includes the ventral tegmental area (VTA) and nucleus accumbens (NAc)^[1,2]^ and may be related to drug addiction.^[3–6]^ Also, orexins modulate dopaminergic, GABAergic, glutamatergic, and cholinergic neurons.^[7–9]^

Orexins (hypocretins) including orexin-A and orexin-B are neuropeptides synthesized by neurons in the lateral and dorsomedial hypothalamus and perifornical areas.^[10,11]^ Two orexin receptors (OX1R and OX2R) mediated with orexins A and B can trigger different effects. In addition, a higher affinity for OX1R was found in orexin-A than in orexin-B. However, OX2R has similar affinities for both orexins A and B.^[12–14]^ Some studies have shown that OX2R plays a role in the regulation of energy homeostasis and the sleep/wake cycle,^[15–17]^ while OX1R modulates reward-seeking and motivated behavior.^[18,19]^ Previous reports showed that orexin-A is involved in drug-seeking.^[18,20]^

Harm reduction therapy might play a role in risky and injected substance use.^[21]^ Methadone maintenance treatment (MMT), frequently used in harm reduction, might increase orexin-A levels in heroin use disorder (HUD) patients.^[22]^

Brain-derived neurotrophic factor (BDNF) a brain neurotrophin may be related to drug addiction severity^[23]^ and may even regulate drug addiction-related behavior.^[24–26]^ Some reports have shown that BDNF levels are lower in substance-dependent subjects than in healthy controls (HCs).^[23,27,28]^

Numerous MRI studies have shown that chronic drug exposure may be associated with a smaller frontal cortex volume, as well as with cognitive and decision-making problems in drug abusers,^[29–31]^ enlarged basal ganglia,^[32–34]^ and more extensive gray matter deficit in the cingulate, limbic, and paralimbic cortices^[35]^ than in normal subjects.^[36]^ Some reports showed that the persistent craving for drugs may be positively correlated with activity in the NAc, inferior frontal/orbitofrontal gyrus, and anterior cingulate.^[37–40]^ Previous study has also revealed that some brain regions regulate reward systems that are associated with treatment response in subjects undergoing addiction intervention.^[41]^

Some research results suggest that gray matter reduction in the bilateral hypothalamus, thalamus, NAc, anterior cingulate cortex, left mid-orbital and rectal gyri, right inferior frontal, and superior temporal gyri may be related to the orexin pathway in narcolepsy.^[42]^ However, there are few reports of the correlation of orexin levels with the functional brain network in HUD patients undergoing harm reduction treatment (HRD). Therefore, the aim of this study was an investigation of changes in serum levels of orexin-A and the functional brain network in HUD patients undergoing harm reduction therapy (HRT).

## 2. Method

### 2.1. Patients and study design

Twenty-five HUD (22 males, 3 females) patients who were undergoing HRT at the Kaohsiung Chang Gung Memorial Hospital were enrolled as subjects for the study. Twenty-four of them were on methadone maintenance treatment and 1 was undergoing buprenorphine therapy. Inclusion criteria were as follows:

1. A diagnosis of HUD based on DSM-5 (The Diagnostic and Statistical Manual of Mental Disorders, Fifth Edition);
2. Age from 20 to 65 years;
3. No history of psychotic, bipolar, major depressive, or substance use disorder except heroin or nicotine use;
4. Seronegative for human immunodeficiency virus; and
5. Stable physical condition. Data collected included age, body mass index (kg/m^2^), methadone or buprenorphine dose, onset age, duration of HRT, serum orexin-A, and BDNF levels. A single board-certified psychiatrist made the diagnosis of HUD for each participant. Blood was drawn from each subject during the no-heroin withdrawal period.

Thirty-one HC subjects (27 males, 4 females) were also recruited. No subjects with any medical or mental disorder were included. All were healthy and none were taking medication.

All participants underwent functional brain MRI scans to compare the functional brain networks of the 2 groups. All participants signed informed consent agreements after the aims and procedures of the study had been explained to them. This study had the prior approval of the Institutional Review Board of the hospital (IRB number: 201801378A0C601A3) and was carried out at the Kaohsiung Chang Gung Memorial Hospital in accordance with the Declaration of Helsinki and Good Clinical Practice guidelines.

### 2.2. Laboratory data

Firstly, blood samples (15 mL) were drawn from a forearm vein of each subject after a fast of at least 8 hours. The samples were immediately centrifuged at 3000*g* for 10 minutes to separate the serum which was stored at −80°C (1–3 months) for analysis. Enzyme-linked immunosorbent assays were carried out using commercially available assay kits for orexin-A (MyBioSource) and BDNF (Promega Corporation, Wisconsin, WI). One trained laboratory technician performed all the analyses in the same laboratory.

### 2.3. MR image acquisition and processing

Functional imaging data were acquired using a 3.0 T GE Signa MRI scanner (Milwaukee, WI). Resting state images were gathered using an echo planar imaging (EPI) sequence (repetition time: 2500 ms; echo time: 27 ms; field of view: 1540 × 1540 mm; flip angle: 77°; matrix size: 64 × 64; slice: 3.4 mm). For the resting state experiment, the scanner room was darkened and the subjects were required to relax, with their eyes closed, and to think of nothing without falling asleep. 3D T1-weighted anatomic images were obtained using an inversion recovery fast spoiled gradient-recalled echo pulse sequence (repetition time: 25 ms; echo time: 7.5 ms; flip angle: 24°; field of view: 192 × 256 mm; matrix size: 288 × 384).

### 2.4. Image preprocessing of rs-fMRI dataset

Preprocessing of resting state functional MRI datasets was carried out using the FMRIB Software Library.^[43,44]^ The following standard preprocessing pipeline was applied for each subject:

1. Removal of the first 10 volumes from the whole-time series to allow for T1-equilibration effects;
2. Correction of temporal shifts in rs-fMRI data acquisition (slice timing correction);
3. Realignment of the rs-fMRI data to the first volume of the whole-time series dataset using MCFLIRT (the FMRIB Motion Correction Linear Image Registration Tool);^[45]^
4. Removal of non-brain tissue using the BET Brain Extraction Tool;^[46]^
5. Spatial smoothing with a 6 mm full width at half maximum Gaussian kernel;
6. Grand–mean intensity normalization;
7. Band-pass temporal filtering (0.01–0.1 Hz) to remove low-frequency drift and high-frequency noise; and
8. Removal of nuisance signals to minimize nonneural noise (head motion, white matter, and cerebrospinal fluid [CSF] signals).

In addition, 3-dimensional rigid-body motion correction (realigned to the first EPI acquisition) was carried out using MCFLIRT. Any subject showing a maximum rotation of 2° or displacement of 2 mm in any direction was excluded from further analysis. To ensure that the final functional connectivity (FC) results were not biased by head motion, the 3 rotational and translational displacement parameters from MCFLIRT were also used to calculate the mean frame displacement of each subject, the criterion was 0.5 mm. After preprocessing, residual RS-fMRI datasets were entered into the Montreal Neurological Institute (MNI) EPI template space and interpolated to a voxel size of 2 m^3^ (2 × 2 × 2). The preprocessed RS-fMRI data were used in the following seed-based FC analysis.

### 2.5. Seed-based functional connectivity

**Figure 1.**
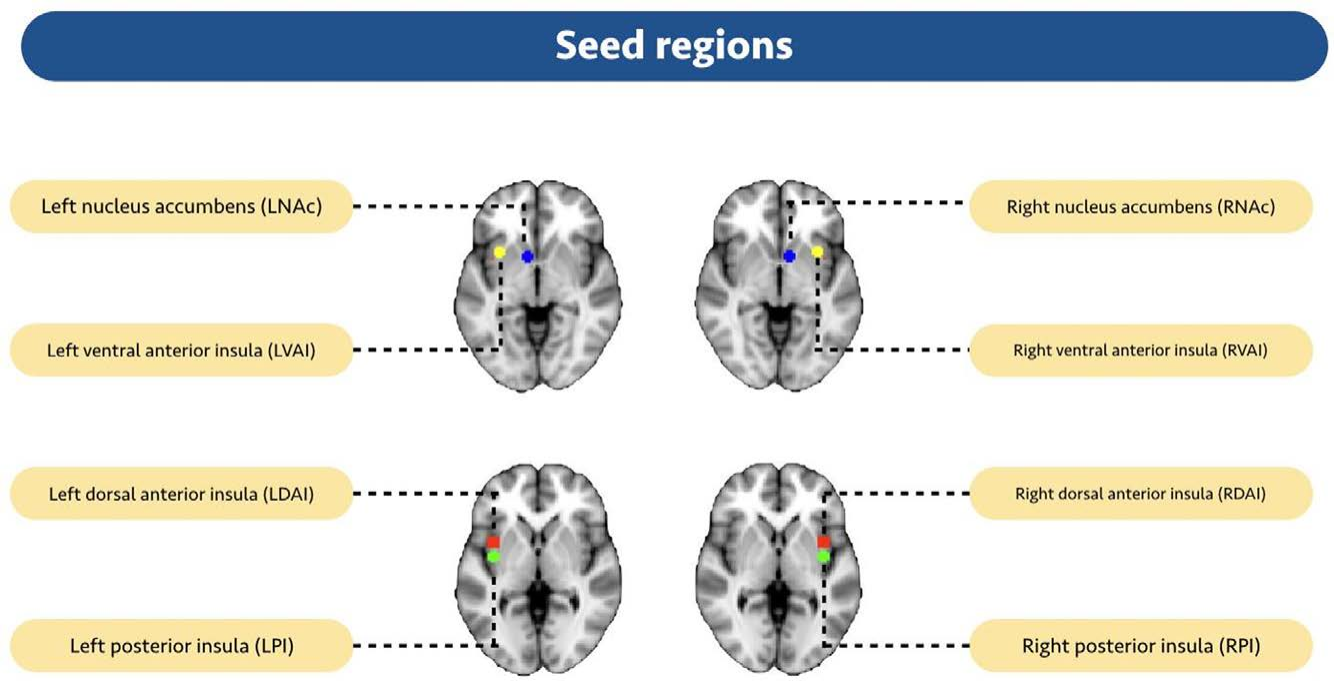
Functional brain network seed points. Four areas in the bilateral brain were defined as seed points. LDAI = left dorsal anterior insula, LNac = left nucleus accumbens, LPI = left posterior insula, LVAI = left ventral anterior insula, RDAI = right dorsal anterior insula, RNac = right nucleus accumbens, RPI = right posterior insula, RVAI = right ventral anterior insula.

To compute the resting state FC of the insula and NAc, 8 spherical seed regions of interest (radius = 6 mm) were defined using previously published NAc and insular subdivisions,^[47,48]^ each corresponding to the left and right NAc (MNI coordinates: −8, 8, −8 and 10, 8, −8), the left and right ventral anterior insula (MNI coordinates: −33, 13, −7 and 32, 10, −6), the left and right dorsal anterior insula (MNI coordinates: −38, 6, 2 and 35, 7, 3), and the left and right posterior insula (MNI coordinates: −38, −6, 5 and 35, −11, 6, see Fig. 1).

Pearson’s correlation analysis was carried out for between-group comparisons of seed time series and the time series of all voxels in the brain for each subject. To improve the normality of the correlation coefficients, Fisher’s *r*-to-*z* transformation was used to convert correlation maps to *Z*-value maps. Group analyses were performed for the correlation maps of each seed region. The correlation maps of HC and HUD were *t* tested and used separately to demonstrate brain regions with significant positive correlations with the NAc and subregions of insula, see Figure S1, Supplemental Digital Content, http://links.lww.com/MD/H13.

### 2.6. Within network connectivity analysis

For between-group comparisons, all individual subject correlation maps were subjected to analysis of covariance with age, sex, and mean FD as covariates (*P* < .05, Family-Wise Error [FWE] corrected).

### 2.7. Between network connectivity analysis

Pearson’s correlation analysis was used to compute the correlation coefficient among the correlation maps which had been derived from the 8 seed regions. For normality correlation, coefficients were transformed to *z*-scores using Fisher’s *r*-to-*z* transformation. The correlation coefficients were then used in an analysis of covariance with age, sex, and mean FD as covariates to compare difference of FC between the groups (*P* < .05, FWE corrected).

### 2.8. Statistical analysis

The results were expressed as the mean ± standard deviation. Comparisons between the orexin-A and BDNF in both HUD patients undergoing HRT and the control group were assessed using the *t* test. Pearson correlation was used to estimate the relationships between the FC of brain networks, and orexin-A and BDNF in patients with HUD. A *P* value of <0.05 was considered statistically significant.

## 3. Results

### 3.1. Characteristics of the included sample

The samples included 25 HUD patients undergoing HRT and 31 healthy subjects.

Table 1 shows the demographic data of the HUD patients that underwent HRT. The average age of the subjects was 46.03 ± 4.38 years, their average body mass index was 25.11 ± 4.22, average age at first time heroin use was 23.24 ± 7.1, the average dose of methadone was 58.12 ± 29.8 mg per day, and average length of treatment was 38.22 ± 38.8 months.

**Table 1:** Demographic data of HUD patients undergoing harm reduction therapy.

Variable	Patients (n = 25)
Age (yr)	46.03 ± 4.38
Sex	22 males, 3 females
BMI (kg/m^2^)	25.11 ± 4.22
Age at first time of heroin use (yr)	23.24 ± 7.1
Methadone dosage in a recent single month (mg/d) (n = 1, buprenorphine 2 mg/d)	58.12 ± 29.8
Duration (mo) of harm reduction therapy	38.22 ± 38.8

Plus-minus values are given as mean ± standard deviation.

BMI = body mass index, HUD = heroin use disorder.

### 3.2. Orexin-A level in HUD patients

The (*t* tested) serum levels of orexin-A were significantly higher in HUD patients undergoing HRT than in the HC (*P* < .001). However, changes in BDNF levels were insignificant, see Table 2.

**Table 2:** Orexin-A and BDNF of HUD and healthy controls.

Variable	Patients (n = 25)	Controls (n = 31)	*t* Test
Age (yr)	46.03 ± 4.38	49.35 ± 10.06	
BMI (kg/m^2^)	25.11 ± 4.22	25.66 ± 3.82	
Orexin-A (pg/mL)	537.95 ± 123.8	404.21 ± 99.63	*P* < .001
BDNF conc (pg/mL)	20,709.93 ± 6326.21	21,676.58 ± 4698.65	*P* = .528

Plus-minus values are given as mean ± standard deviation.

BDNF = brain-derived neurotrophic factor, BMI = body mass index, HUD = heroin use disorder.

### 3.3. Functional connectivity between groups

**Figure 2.**
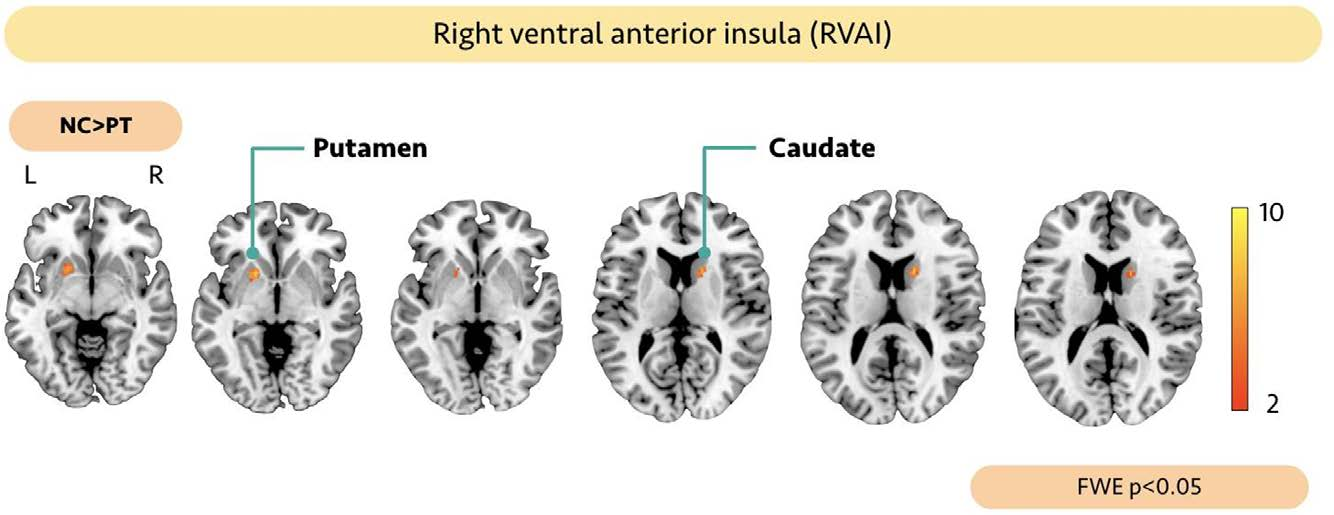
Group comparison of RVAI functional network. The HUD group subjects showed significantly lower FC in the right caudate (*x*, *y*, *z* = 16, 12, 14) and left putamen (*x*, *y*, *z* = −18, 12, −4) in the RVAI functional network (*P* < .05, FWE corrected) than those of the control group. FC = functional connectivity, FWE = Family-Wise Error, HUD = heroin use disorder, RVAI = right ventral anterior insula.

Figure 2 shows the difference in intranetwork FC between the groups. HUD subjects showed significantly lower FC in the right ventral anterior insula functional network (*P* < .05, FWE corrected) than those in the HC group. However, no significant differences were observed in the intranetwork FC in left ventral anterior insula, bilateral NAc, bilateral dorsal anterior insula, and bilateral posterior insula, between the 2 groups.

**Figure 3.**
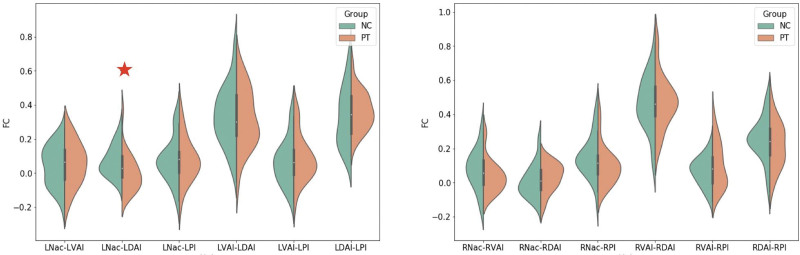
Group comparison of interaction functional networks among seed points. The HUD group subjects showed significantly lower FC between LNAc and LDAI (*P* < .05, FWE corrected) than those in the control group. FC = functional connectivity, FWE = Family-Wise Error, HUD = heroin use disorder, LDAI = left dorsal anterior insula, LNac = left nucleus accumbens, LPI = left posterior insula, LVAI = left ventral anterior insula, RDAI = right dorsal anterior insula, RNac = right nucleus accumbens, RPI = right posterior insula, RVAI = right ventral anterior insula.

Figure 3 shows the difference of internetwork FC between the groups and more analyses were conducted to determine internetwork FC differences. These showed that FC between left NAc and left dorsal anterior insula (*P* < .05, FWE corrected) was significantly lower in the HUD group subjects than in those of the HC group.

Further correlations were analyzed to assess the relationship between the level of orexin-A and strength of the network. However, no significant correlation between orexin-A level and the functional brain network was found in this study.

## 4. Discussion

One of important finding in this study was that HUD patients undergoing HRT had significantly higher serum levels of orexin-A than the HC group subjects. In addition, the functional brain networks indicated significant differences in RVAI between 2 groups. Although the correlation between orexin-A and functional brain network was not significant, further study of their association was needed. There may be some undefined reason(s) to explain why the correlation was not significant. The limited sample size could be 1. Orexins commonly exist in CSF and levels fluctuate slightly with the diurnal cycle, where the highest levels are seen in the middle of the night.^[49]^ Orexin is also present in human plasma with an uncertain secretion source^[50]^ and the level may be about 30% lower than that observed in the CSF.^[51]^ In addition, a previous study showed no significant difference in plasma orexin-A level over a period of 1 day.^[50]^ This means that plasma orexin-A level may not be a true reflection of its function in the central nervous system.

As we know, of all the neurotransmitters, dopamine is most closely related to the mechanism of drug addiction. Orexin-A could activate the VTA dopamine neurons by mediating with OX1R and the dopamine would then be released from VTA.^[52]^ A previous study revealed that addictive substances originating from the VTA act on the mesocorticolimbic dopamine system and cause elevated dopamine concentrations in the NAc.^[53]^

It has been shown that the NAc might play a role in addiction-related behavior. The dopamine transmission in the NAc shell could be stimulated by the addictive drug and cause drug-conditioned behavior.^[54]^ Furthermore, the glutamatergic projections within the NAc might regulate drug-seeking and cause a relapse to substance use.^[55]^

Data from the present study suggest that functional RVAI activity in HUD subjects undergoing HRT show less significant differences in the intranetwork in FC than in HC. Another finding was that HUD group subjects showed significantly lower internetwork FC between left NAc and left dorsal anterior insula compared to those in a HC group (*P* < .05).

For years, neuroscience research has supported the idea that the anterior insula is associated with emotional experience,^[56]^ bodily sensation,^[57]^ and affective feelings.^[58]^ A ventral anterior insula region connected with the limbic system participates in affective processes, and a dorsal anterior insula subdivision with connections to frontal, anterior cingulate, and parietal regions is involved in cognitive control processes.^[59–61]^ Some studies highlight the important role of the left anterior insula in social function, such as empathy.^[61–63]^ More recent functional imaging studies of the left anterior insula also present its function in language, especially the motor aspects of speech production.^[64–66]^ The right anterior insula (RAI) might participate in sympathetic nervous system function, because lesions in the RAI may cause elevated heart rate^[67]^ and peripheral noradrenergic transmitter levels.^[68]^

Recent evidence indicates that the insula plays a role in drug addiction.^[69]^ The probable role in conscious urges to take drugs was noted in the insula.^[64,70]^ In addition, elevated RAI activity is seen in subjects with anxiety disorders^[71]^ and might regulate decision making that involves uncertain risk and reward.^[72,73]^ Some reviews indicated that decreased insula activity may cause some of the abnormal decision making that may lead to relapse and further drug use.^[74,75]^ Also, previous research showed decreased gray matter in the insula in substance-dependent patients.^[76,77]^ The left insula was more affected in methamphetamine users, whereas in cocaine users it was the right insula.^[78]^ Those findings were consistent with our results showing lower FC in RAVI in HUD patients undergoing HRT than in HC.

Our study was limited by sample size. It was also not clear if there was a significant correlation between orexin-A and the functional brain network. Future study will involve far more subjects.

## 5. Conclusions

In conclusion, the orexin-A levels in HUD subjects undergoing HRT showed significantly greater differences than those in the HC group. However, the functional brain network showed lower FC in HUD patients undergoing HRT than in HC. Our results suggest that HRT might increase orexin-A levels and decrease functional activity in RVAI in the HUD subjects.

## Author contributions

**Conceptualization:** Meng-Chang Tsai.

**Formal analysis:** Meng-Chang Tsai, Wei-Che Lin.

**Investigation:** Meng-Chang Tsai.

**Methodology:** Meng-Chang Tsai, Liang-Chun Lin.

**Project administration:** Meng-Chang Tsai.

**Resources:** Meng-Chang Tsai, Liang-Chun Lin.

**Supervision:** Meng-Chang Tsai.

**Validation:** Meng-Chang Tsai, Liang-Chun Lin.

**Visualization:** Liang-Chun Lin.

**Writing – original draft:** Wan-Ru You.

**Writing – review & editing:** Meng-Chang Tsai, Wei-Che Lin.

## Supplementary Material



## References

[1] Sakurai T. The role of orexin in motivated behaviours. Nat Rev Neurosci. 2014;15:719–31. 25301357 10.1038/nrn3837

[2] Wise RA Rompre PP. Brain dopamine and reward. Annu Rev Psychol. 1989;40:191–225. 2648975 10.1146/annurev.ps.40.020189.001203

[3] Farahimanesh S Zarrabian S Haghparast A. Role of orexin receptors in the ventral tegmental area on acquisition and expression of morphine-induced conditioned place preference in the rats. Neuropeptides. 2017;66:45–51. 28890208 10.1016/j.npep.2017.08.003

[4] Ziolkowski M Czarnecki D Budzynski J. Orexin in patients with alcohol dependence treated for relapse prevention: a pilot study. Alcohol Alcohol. 2016;51:416–21. 26597795 10.1093/alcalc/agv129

[5] Bernstein DL Badve PS Barson JR. Hypocretin receptor 1 knockdown in the ventral tegmental area attenuates mesolimbic dopamine signaling and reduces motivation for cocaine. Addict Biol. 2018;23:1032–45. 28971565 10.1111/adb.12553 PMC5880744

[6] Aston-Jones G Smith RJ Moorman DE. Role of lateral hypothalamic orexin neurons in reward processing and addiction. Neuropharmacology. 2009;56(Suppl 1):112–21. 18655797 10.1016/j.neuropharm.2008.06.060 PMC2635332

[7] Arrigoni E Mochizuki T Scammell TE. Activation of the basal forebrain by the orexin/hypocretin neurones. Acta Physiol (Oxf). 2010;198:223–35. 19723027 10.1111/j.1748-1716.2009.02036.x PMC2938067

[8] Balcita-Pedicino JJ Sesack SR. Orexin axons in the rat ventral tegmental area synapse infrequently onto dopamine and gamma-aminobutyric acid neurons. J Comp Neurol. 2007;503:668–84. 17559101 10.1002/cne.21420

[9] Fadel J Frederick-Duus D. Orexin/hypocretin modulation of the basal forebrain cholinergic system: insights from in vivo microdialysis studies. Pharmacol Biochem Behav. 2008;90:156–62. 18281084 10.1016/j.pbb.2008.01.008

[10] Sakurai T Amemiya A Ishii M. Orexins and orexin receptors: a family of hypothalamic neuropeptides and G protein-coupled receptors that regulate feeding behavior. Cell. 1998;92:573–85. 9491897 10.1016/s0092-8674(00)80949-6

[11] de Lecea L Kilduff TS Peyron C. The hypocretins: hypothalamus-specific peptides with neuroexcitatory activity. Proc Natl Acad Sci USA. 1998;95:322–7. 9419374 10.1073/pnas.95.1.322 PMC18213

[12] Lu XY Bagnol D Burke S. Differential distribution and regulation of OX1 and OX2 orexin/hypocretin receptor messenger RNA in the brain upon fasting. Horm Behav. 2000;37:335–44. 10860677 10.1006/hbeh.2000.1584

[13] Xu TR Yang Y Ward R. Orexin receptors: multi-functional therapeutic targets for sleeping disorders, eating disorders, drug addiction, cancers and other physiological disorders. Cell Signal. 2013;25:2413–23. 23917208 10.1016/j.cellsig.2013.07.025

[14] Trivedi P Yu H MacNeil DJ. Distribution of orexin receptor mRNA in the rat brain. FEBS Lett. 1998;438:71–5. 9821961 10.1016/s0014-5793(98)01266-6

[15] Funato H Tsai AL Willie JT. Enhanced orexin receptor-2 signaling prevents diet-induced obesity and improves leptin sensitivity. Cell Metab. 2009;9:64–76. 19117547 10.1016/j.cmet.2008.10.010 PMC2630400

[16] Dugovic C Shelton JE Aluisio LE. Blockade of orexin-1 receptors attenuates orexin-2 receptor antagonism-induced sleep promotion in the rat. J Pharmacol Exp Ther. 2009;330:142–51. 19363060 10.1124/jpet.109.152009

[17] Sakurai T. The neural circuit of orexin (hypocretin): maintaining sleep and wakefulness. Nat Rev Neurosci. 2007;8:171–81. 17299454 10.1038/nrn2092

[18] Harris GC Wimmer M Aston-Jones G. A role for lateral hypothalamic orexin neurons in reward seeking. Nature. 2005;437:556–9. 16100511 10.1038/nature04071

[19] Hollander JA Lu Q Cameron MD. Insular hypocretin transmission regulates nicotine reward. Proc Natl Acad Sci USA. 2008;105:19480–5. 19033203 10.1073/pnas.0808023105 PMC2614786

[20] Harris GC Wimmer M Randall-Thompson JF. Lateral hypothalamic orexin neurons are critically involved in learning to associate an environment with morphine reward. Behav Brain Res. 2007;183:43–51. 17599478 10.1016/j.bbr.2007.05.025 PMC2030620

[21] Toumbourou JW Stockwell T Neighbors C. Interventions to reduce harm associated with adolescent substance use. Lancet. 2007;369:1391–401. 17448826 10.1016/S0140-6736(07)60369-9

[22] Tsai MC Huang TL. Orexin A in men with heroin use disorder undergoing methadone maintenance treatment. Psychiatry Res. 2018;264:412–5. 29680730 10.1016/j.psychres.2018.04.010

[23] Ornell F Hansen F Schuch FB. Brain-derived neurotrophic factor in substance use disorders: a systematic review and meta-analysis. Drug Alcohol Depend. 2018;193:91–103. 30347311 10.1016/j.drugalcdep.2018.08.036

[24] Benzerouk F Gierski F Raucher-Chene D. Association study between reward dependence and a functional BDNF polymorphism in adult women offspring of alcohol-dependent probands. Psychiatr Genet. 2015;25:208–11. 26204172 10.1097/YPG.0000000000000099

[25] Zimmermann KS Yamin JA Rainnie DG. Connections of the mouse orbitofrontal cortex and regulation of goal-directed action selection by brain-derived neurotrophic factor. Biol Psychiatry. 2017;81:366–77. 26786312 10.1016/j.biopsych.2015.10.026 PMC4871791

[26] Pitts EG Taylor JR Gourley SL. Prefrontal cortical BDNF: a regulatory key in cocaine- and food-reinforced behaviors. Neurobiol Dis. 2016;91:326–35. 26923993 10.1016/j.nbd.2016.02.021 PMC4913044

[27] Angelucci F Ricci V Pomponi M. Chronic heroin and cocaine abuse is associated with decreased serum concentrations of the nerve growth factor and brain-derived neurotrophic factor. J Psychopharmacol. 2007;21:820–5. 17715210 10.1177/0269881107078491

[28] Zhang K Jiang H Zhang Q. Brain-derived neurotrophic factor serum levels in heroin-dependent patients after 26 weeks of withdrawal. Compr Psychiatry. 2016;65:150–5. 26774004 10.1016/j.comppsych.2015.11.010

[29] Liu X Matochik JA Cadet JL. Smaller volume of prefrontal lobe in polysubstance abusers: a magnetic resonance imaging study. Neuropsychopharmacology. 1998;18:243–52. 9509492 10.1016/S0893-133X(97)00143-7

[30] Schlaepfer TE Lancaster E Heidbreder R. Decreased frontal white-matter volume in chronic substance abuse. Int J Neuropsychopharmacol. 2006;9:147–53. 16004619 10.1017/S1461145705005705

[31] Kim SJ Lyoo IK Hwang J. Prefrontal grey-matter changes in short-term and long-term abstinent methamphetamine abusers. Int J Neuropsychopharmacol. 2006;9:221–8. 15982446 10.1017/S1461145705005699

[32] Jacobsen LK Giedd JN Gottschalk C. Quantitative morphology of the caudate and putamen in patients with cocaine dependence. Am J Psychiatry. 2001;158:486–9. 11229995 10.1176/appi.ajp.158.3.486

[33] Chang L Cloak C Patterson K. Enlarged striatum in abstinent methamphetamine abusers: a possible compensatory response. Biol Psychiatry. 2005;57:967–74. 15860336 10.1016/j.biopsych.2005.01.039 PMC4899039

[34] Jernigan TL Gamst AC Archibald SL. Effects of methamphetamine dependence and HIV infection on cerebral morphology. Am J Psychiatry. 2005;162:1461–72. 16055767 10.1176/appi.ajp.162.8.1461

[35] Thompson PM Hayashi KM Simon SL. Structural abnormalities in the brains of human subjects who use methamphetamine. J Neurosci. 2004;24:6028–36. 15229250 10.1523/JNEUROSCI.0713-04.2004 PMC6729247

[36] Fowler JS Volkow ND Kassed CA. Imaging the addicted human brain. Sci Pract Perspect. 2007;3:4–16. 17514067 10.1151/spp07324 PMC2851068

[37] Breiter HC Gollub RL Weisskoff RM. Acute effects of cocaine on human brain activity and emotion. Neuron. 1997;19:591–611. 9331351 10.1016/s0896-6273(00)80374-8

[38] Breiter HC Rosen BR. Functional magnetic resonance imaging of brain reward circuitry in the human. Ann N Y Acad Sci. 1999;877:523–47. 10415669 10.1111/j.1749-6632.1999.tb09287.x

[39] Kufahl PR Li Z Risinger RC. Neural responses to acute cocaine administration in the human brain detected by fMRI. Neuroimage. 2005;28:904–14. 16061398 10.1016/j.neuroimage.2005.06.039

[40] Risinger RC Salmeron BJ Ross TJ. Neural correlates of high and craving during cocaine self-administration using BOLD fMRI. Neuroimage. 2005;26:1097–108. 15886020 10.1016/j.neuroimage.2005.03.030

[41] Hammond CJ Allick A Rahman N. Structural and functional neural targets of addiction treatment in adolescents and young adults: a systematic review and meta-analysis. J Child Adolesc Psychopharmacol. 2019;29:498–507. 31313938 10.1089/cap.2019.0007 PMC6727475

[42] Weng HH Chen CF Tsai YH. Gray matter atrophy in narcolepsy: an activation likelihood estimation meta-analysis. Neurosci Biobehav Rev. 2015;59:53–63. 25825285 10.1016/j.neubiorev.2015.03.009

[43] Smith SM Jenkinson M Woolrich MW. Advances in functional and structural MR image analysis and implementation as FSL. Neuroimage. 2004;23(Suppl 1):S208–19. 15501092 10.1016/j.neuroimage.2004.07.051

[44] Jenkinson M Beckmann CF Behrens TE. Fsl. Neuroimage. 2012;62:782–90. 21979382 10.1016/j.neuroimage.2011.09.015

[45] Jenkinson M Bannister P Brady M. Improved optimization for the robust and accurate linear registration and motion correction of brain images. Neuroimage. 2002;17:825–41. 12377157 10.1016/s1053-8119(02)91132-8

[46] Smith SM. Fast robust automated brain extraction. Hum Brain Mapp. 2002;17:143–55. 12391568 10.1002/hbm.10062 PMC6871816

[47] Zou F Wu X Zhai T. Abnormal resting-state functional connectivity of the nucleus accumbens in multi-year abstinent heroin addicts. J Neurosci Res. 2015;93:1693–702. 26280556 10.1002/jnr.23608

[48] Li XB Wang LB Xiong YB. Altered resting-state functional connectivity of the insula in individuals with clinical high-risk and patients with first-episode schizophrenia. Psychiatry Res. 2019;282:112608. 31655405 10.1016/j.psychres.2019.112608

[49] Salomon RM Ripley B Kennedy JS. Diurnal variation of cerebrospinal fluid hypocretin-1 (Orexin-A) levels in control and depressed subjects. Biol Psychiatry. 2003;54:96–104. 12873798 10.1016/s0006-3223(02)01740-7

[50] Arihara Z Takahashi K Murakami O. Immunoreactive orexin-A in human plasma. Peptides. 2001;22:139–42. 11179609 10.1016/s0196-9781(00)00369-7

[51] Dalal MA Schuld A Haack M. Normal plasma levels of orexin A (hypocretin-1) in narcoleptic patients. Neurology. 2001;56:1749–51. 11425946 10.1212/wnl.56.12.1749

[52] Baimel C Lau BK Qiao M. Projection-target-defined effects of orexin and dynorphin on VTA dopamine neurons. Cell Rep. 2017;18:1346–55. 28178514 10.1016/j.celrep.2017.01.030

[53] Di Chiara G Imperato A. Drugs abused by humans preferentially increase synaptic dopamine concentrations in the mesolimbic system of freely moving rats. Proc Natl Acad Sci USA. 1988;85:5274–8. 2899326 10.1073/pnas.85.14.5274 PMC281732

[54] Di Chiara G Bassareo V Fenu S. Dopamine and drug addiction: the nucleus accumbens shell connection. Neuropharmacology. 2004;47(Suppl 1):227–41. 15464140 10.1016/j.neuropharm.2004.06.032

[55] Scofield MD Heinsbroek JA Gipson CD. The nucleus accumbens: mechanisms of addiction across drug classes reflect the importance of glutamate homeostasis. Pharmacol Rev. 2016;68:816–71. 27363441 10.1124/pr.116.012484 PMC4931870

[56] Lindquist KA Wager TD Kober H. The brain basis of emotion: a meta-analytic review. Behav Brain Sci. 2012;35:121–43. 22617651 10.1017/S0140525X11000446 PMC4329228

[57] Craig AD. How do you feel? Interoception: the sense of the physiological condition of the body. Nat Rev Neurosci. 2002;3:655–66. 12154366 10.1038/nrn894

[58] Craig AD. How do you feel-now? The anterior insula and human awareness. Nat Rev Neurosci. 2009;10:59–70. 19096369 10.1038/nrn2555

[59] Nomi JS Farrant K Damaraju E. Dynamic functional network connectivity reveals unique and overlapping profiles of insula subdivisions. Hum Brain Mapp. 2016;37:1770–87. 26880689 10.1002/hbm.23135 PMC4837017

[60] Glasser MF Coalson TS Robinson EC. A multi-modal parcellation of human cerebral cortex. Nature. 2016;536:171–8. 27437579 10.1038/nature18933 PMC4990127

[61] Uddin LQ Nomi JS Hebert-Seropian B. Structure and Function of the Human Insula. J Clin Neurophysiol. 2017;34:300–6. 28644199 10.1097/WNP.0000000000000377 PMC6032992

[62] Papagno C Pisoni A Mattavelli G. Specific disgust processing in the left insula: new evidence from direct electrical stimulation. Neuropsychologia. 2016;84:29–35. 26836143 10.1016/j.neuropsychologia.2016.01.036

[63] Lerner JS Li Y Valdesolo P. Emotion and decision making. Annu Rev Psychol. 2015;66:799–823. 25251484 10.1146/annurev-psych-010213-115043

[64] Naqvi NH Bechara A. The hidden island of addiction: the insula. Trends Neurosci. 2009;32:56–67. 18986715 10.1016/j.tins.2008.09.009 PMC3698860

[65] Ackermann H Riecker A. The contribution of the insula to motor aspects of speech production: a review and a hypothesis. Brain Lang. 2004;89:320–8. 15068914 10.1016/S0093-934X(03)00347-X

[66] Shuren J. Insula and aphasia. J Neurol. 1993;240:216–8. 8496709 10.1007/BF00818707

[67] Abboud H Berroir S Labreuche J. Insular involvement in brain infarction increases risk for cardiac arrhythmia and death. Ann Neurol. 2006;59:691–9. 16566012 10.1002/ana.20806

[68] Meyer S Strittmatter M Fischer C. Lateralization in autonomic dysfunction in ischemic stroke involving the insular cortex. Neuroreport. 2004;15:357–61. 15076768 10.1097/00001756-200402090-00029

[69] Naqvi NH Rudrauf D Damasio H. Damage to the insula disrupts addiction to cigarette smoking. Science. 2007;315:531–4. 17255515 10.1126/science.1135926 PMC3698854

[70] Garavan H Pankiewicz J Bloom A. Cue-induced cocaine craving: neuroanatomical specificity for drug users and drug stimuli. Am J Psychiatry. 2000;157:1789–98. 11058476 10.1176/appi.ajp.157.11.1789

[71] Etkin A Wager TD. Functional neuroimaging of anxiety: a meta-analysis of emotional processing in PTSD, social anxiety disorder, and specific phobia. Am J Psychiatry. 2007;164:1476–88. 17898336 10.1176/appi.ajp.2007.07030504 PMC3318959

[72] Mesulam MM Mufson EJ. Insula of the old world monkey. I. Architectonics in the insulo-orbito-temporal component of the paralimbic brain. J Comp Neurol. 1982;212:1–22. 7174905 10.1002/cne.902120102

[73] Eckert MA Menon V Walczak A. At the heart of the ventral attention system: the right anterior insula. Hum Brain Mapp. 2009;30:2530–41. 19072895 10.1002/hbm.20688 PMC2712290

[74] Ernst M Paulus MP. Neurobiology of decision making: a selective review from a neurocognitive and clinical perspective. Biol Psychiatry. 2005;58:597–604. 16095567 10.1016/j.biopsych.2005.06.004

[75] Paulus MP Tapert SF Schuckit MA. Neural activation patterns of methamphetamine-dependent subjects during decision making predict relapse. Arch Gen Psychiatry. 2005;62:761–8. 15997017 10.1001/archpsyc.62.7.761

[76] Franklin TR Acton PD Maldjian JA. Decreased gray matter concentration in the insular, orbitofrontal, cingulate, and temporal cortices of cocaine patients. Biol Psychiatry. 2002;51:134–42. 11822992 10.1016/s0006-3223(01)01269-0

[77] Ersche KD Williams GB Robbins TW. Meta-analysis of structural brain abnormalities associated with stimulant drug dependence and neuroimaging of addiction vulnerability and resilience. Curr Opin Neurobiol. 2013;23:615–24. 23523373 10.1016/j.conb.2013.02.017

[78] Hall MG Alhassoon OM Stern MJ. Gray matter abnormalities in cocaine versus methamphetamine-dependent patients: a neuroimaging meta-analysis. Am J Drug Alcohol Abuse. 2015;41:290–9. 26125488 10.3109/00952990.2015.1044607

