# Supplementary material for: Differences in orexin-A level in the functional brain network of HUD patients undergoing harm reduction therapy
Source: Medicine (Baltimore). 2022 Aug 19;101(33):e30093. doi: 10.1097/MD.0000000000030093 (PMC9387983; doi:10.1097/MD.0000000000030093)

**Supplementary Figure Legend**

Large-scale functional networks observed using seed-based analyses from 8 seeds in HUD and HC. The shown functional networks were the result of single-sample t- tests (T>3, uncorrected p< 0.05).

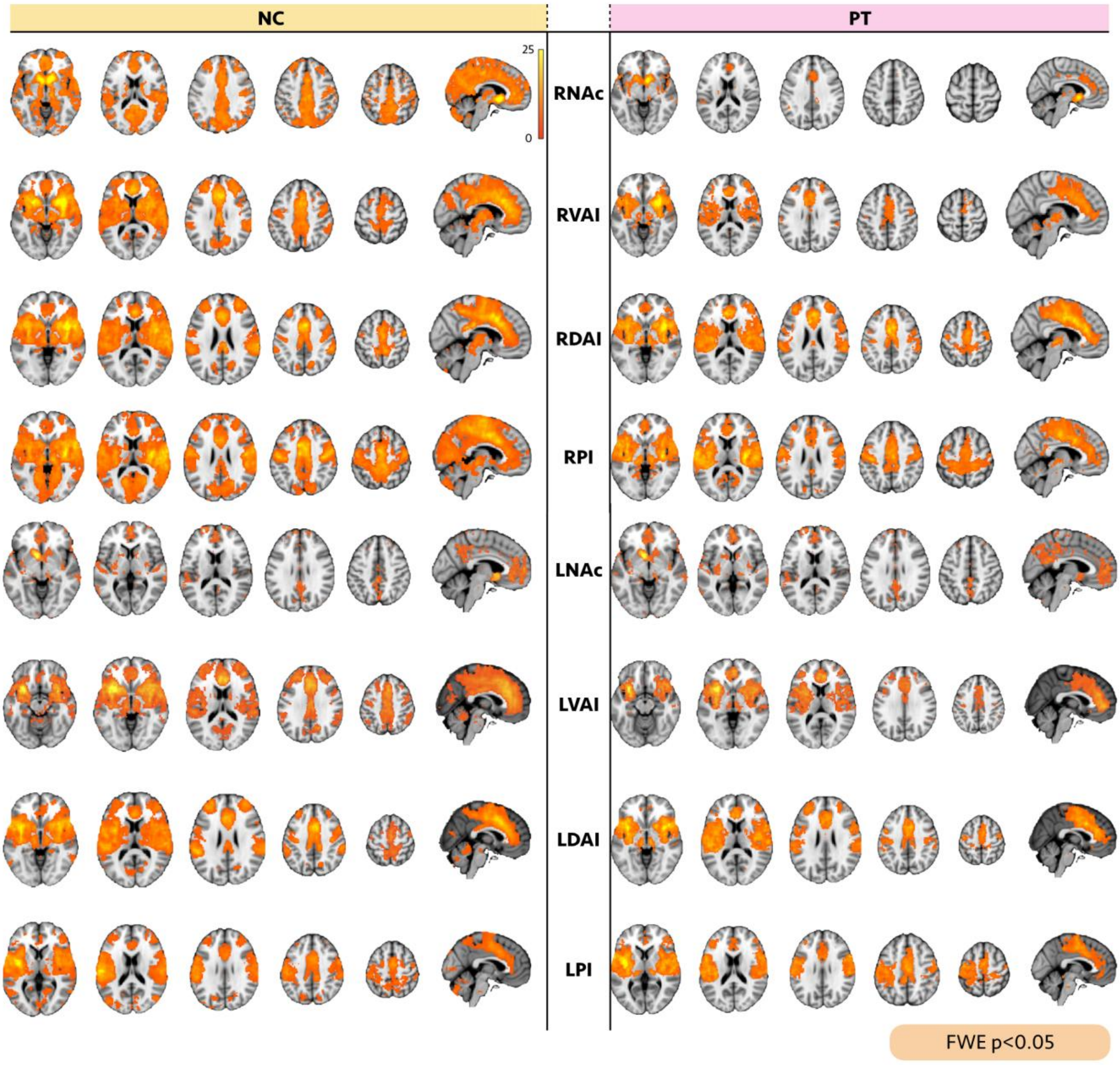

Supplement: Supplementary file 1 [file medi-101-e30093-s001.pdf]
